# Consumption of JUUL vs. Other E-Cigarette Brands among U.S. E-Cigarette Users: Evidence from Wave 5 of the PATH Study

**DOI:** 10.3390/ijerph191710837

**Published:** 2022-08-31

**Authors:** Yu Wang, Zongshuan Duan, Scott R. Weaver, Lucy Popova, Claire A. Spears, David L. Ashley, Terry F. Pechacek, Michael P. Eriksen, Jidong Huang

**Affiliations:** 1 School of Public Health, Georgia State University, Atlanta, GA 30302, USA; 2 Milken Institute School of Public Health, George Washington University, Washington, DC 20052, USA

**Keywords:** JUUL, e-cigarette brands, consumption share, e-cigarette dependence, youth, young adult, adult

## Abstract

This study examines the use of JUUL vs. other e-cigarette brands among U.S. youth (12–17 years), young adult (18–24 years), and adult (25 years and above) e-cigarette users. Data were from the Population Assessment of Tobacco and Health (PATH) study Wave 5 survey (2019). The study population was past 30-day e-cigarette users who knew the brand of e-cigarettes they usually/last used (N = 2569). JUUL use was reported by 65.2% of youth, 60.7% of young adult, and 25.6% of adult e-cigarette users in our study sample. The share of JUUL consumed in the past 30 days, measured by the total number of puffs, was 15.4% by youth, 55.5% by young adults, and 29.1% by adults. By contrast, the share of other e-cigarettes consumed was 4.2% by youth, 28.9% by young adults, and 66.9% by adults. Youth JUUL users were more likely to use e-cigarettes within 30 min after waking (aOR = 2.30, 95% CI: 1.12–4.75) than youth users of other brands of e-cigarettes. Additionally, youth e-cigarette users who currently smoked cigarettes were less likely to use JUUL (aOR = 0.55, 95% CI: 0.30–0.99). This study concludes that JUUL consumption was disproportionally higher among youth and young adults in the U.S. in 2019.

## 1. Introduction

JUUL, a USB drive-shaped, rechargeable e-cigarette device with a disposable prefilled pod system, has gained considerable market share since it entered the United States (U.S.) market in 2015 [1,2,3,4,5]. Evidence demonstrated that JUUL e-cigarettes were particularly popular among youth in the U.S. [3,6,7]. Data from the National Youth Tobacco Survey (NYTS) showed that the prevalence of current (past 30-day) e-cigarette use increased significantly from 2017 to 2019 among middle school students (3.3% to 10.5%) and high school students (11.7% to 27.5%) [8,9], which coincided with the expansion of JUUL marketing and the increased sales of JUUL e-cigarettes [1,10,11]. In December 2018, the U.S. Surgeon General declared youth vaping an epidemic and called out JUUL as part of the problem [4]. The 2019 NYTS reported that among current e-cigarette users, 59.1% of high school students and 54.1% of middle school students reported JUUL as their usual e-cigarette brand [9]. According to the 2020 and 2021 NYTS data, e-cigarette use may have declined among U.S. youth (high school students: 19.6% in 2020, 11.3% in 2021; middle school students: 4.7% in 2020, 2.8% in 2021) [12,13]. However, this decline may be due to the changes in NYTS survey design and mode (from school-based in-person surveys to online surveys) as a result of the COVID-19 pandemic. Indeed, the Centers for Disease Control and Prevention (CDC) advised not to compare results from the 2020/2021 NYTS with findings from previous surveys [12,13]. Still, more than 2 million U.S. youth were using e-cigarettes in 2021, and JUUL remains the fourth most popular brand among high school students and the second most popular brand among middle school students in the U.S. [12].

Previous studies indicated that the popularity of JUUL among youth may be driven, at least partially, by its product design, use of nicotine salt, product flavors, and marketing [1,14,15,16,17]. JUUL’s product design, including minimal visible exhaled aerosol, reduced odor, and small size, makes it easier to conceal from parents and teachers and to be used discreetly [16]. In addition, JUUL was noted for its innovative method of delivering nicotine [17]. With its patented nicotine salt technology using benzoic acid, JUUL delivers a high nicotine concentration less harshly and bitterly [18,19]. Previous studies demonstrated that JUUL’s peak nicotine levels were comparable to or greater than combustible cigarettes for experienced e-cigarette users [20,21]. Jackler and Ramamurthi (2019) found that the popularity of JUUL triggered the marketing of a wide variety of pod-based e-cigarettes and e-liquids with exceptionally high nicotine levels [22]. In addition, JUUL pods initially offered a variety of youth-appealing flavors, such as crème brulee, mango, and cucumber, which were discontinued for sale in late 2018 due to intense regulatory scrutiny [23]. Furthermore, evidence suggests that JUUL’s aggressive and youth-targeted social media marketing contributed to its popularity among youth in the U.S. [1,5,24] and led to greater nicotine addiction among youth [25,26]. This raised concerns about the risks of JUUL use among youth and prompted regulatory actions targeted toward JUUL. In 2018, the U.S. Food and Drug Administration (FDA) ordered JUUL to submit documents regarding its marketing practices due to concerns about the popularity of JUUL among youth [27]. In 2019, congressional hearings were held to examine JUUL’s role in the youth e-cigarette epidemic [28].

The number of lawsuits against JUUL Labs, Inc. has steadily increased in the past two years [29]. Many lawsuits accused JUUL marketed its products in a manner to attract minors, and the company promoted nicotine use among youth [29,30]. However, JUUL has denied such accusations and instead claims that its marketing had primarily focused on adults and that its products are designed to help adult smokers switch from cigarettes to e-cigarettes [31]. On 23 June 2022, the FDA announced its denial of authorization to market JUUL products because “the FDA determined that JUUL applications lacked sufficient evidence regarding the toxicological profile of the products to demonstrate that marketing of the products would be appropriate for the protection of the public health” [32]. The FDA also highlighted JUUL’s “disproportionate role in the rise in youth vaping” in its marketing denial order [32]. Immediately after the FDA’s announcement, JUUL Labs, Inc. challenged FDA’s denial order in a federal court. On 24 June 2022, the U.S. Court of Appeals for the D.C. Circuit entered a temporary administrative stay of the marketing denial order for Juul Labs Inc. [32]. Subsequently, on 5 July 2022, the FDA issued an administrative stay on the marketing denial order while conducting further reviews on the scientific issues unique to the JUUL Premarket Tobacco Product Application (PMTA) [32].

A key issue central to legal cases against JUUL is to what extent JUUL e-cigarettes are used by youth. Consequently, estimating the share of JUUL consumed by each age group is critically important in evaluating the company’s marketing claims, which could provide important evidence to help inform ongoing legal cases and the FDA’s review of JUUL. However, data on the share of JUUL consumed by youth, young adults, and adults are scarce. Kaplan et al. (2021) used data from the Population Assessment of Tobacco and Health (PATH) study Wave 4 and found that 10–50% of JUUL’s net revenue was from youth in 2017 [33]. However, even with its large sample size, only 25 youth participants and 34 adult participants reported using JUUL in the PATH Wave 4 survey. As such, the estimated prevalence of JUUL use had wide confidence intervals and may be subject to biases.

Studies investigating factors associated with the use of JUUL vs. other e-cigarette brands are also rare. Vallone et al. (2019) found that younger age, being white, having better financial resources, and current combustible tobacco use was associated with JUUL use [6]. However, this study compared JUUL users with the general population instead of users of other e-cigarette brands. Additionally, although previous studies have documented that JUUL could deliver nicotine more efficiently than standard freebase nicotine e-cigarettes, and a large proportion of youth JUUL users reported symptoms of nicotine dependence [22,25,34], few studies have examined e-cigarette dependence among JUUL users compared with users of other brands. Mantey et al. (2021) found that JUUL users were more likely to report frequent e-cigarette use and nicotine dependence symptoms compared with non-JUUL users among U.S. middle and high school students [35]. However, the association between e-cigarette dependence and the use of JUUL vs. other brands has not been examined for other age groups.

Importantly, previous studies primarily focused on the prevalence of JUUL use and overlooked the intensity of use (consumption level) of JUUL vs. other e-cigarette brands [3,33,35]. It is possible that the prevalence of JUUL use may be higher among youth and young adults than adults; however, if JUUL were used more frequently and intensively among adults compared to youth, the share of total JUUL consumption could still be higher among adults.

This study aims to address these critical gaps in the literature by comparing the use of JUUL vs. other e-cigarette brands from the perspective of consumption shares, use frequency, and associations with e-cigarette dependence among youth, young adult, and adult e-cigarette users in the US.

## 2. Materials and Methods

### 2.1. Data

This study used data from the Wave 5 survey of the PATH study, a large ongoing longitudinal study representative of the non-institutionalized U.S. population 12 years and older [36]. The PATH study is a collaboration between the U.S. National Institutes of Health (NIH) and the FDA. The Wave 5 survey was conducted from December 2018 to November 2019. Detailed study design, sampling strategies, and guidelines to download and use the PATH data/documentation files can be found in previously published studies [36,37]. The target population of this study was past 30-day e-cigarette users who knew the brand name of e-cigarettes they usually/last used. In the PATH study, past 30-day e-cigarette users were asked, “Do/Did you have a regular brand of electronic nicotine product/electronic nicotine cartridge/e-liquid you usually use/used?” Respondents who answered “Yes” were asked if they knew the name of the brand they usually used. Those who did not report the names of the brands they usually used were asked if they knew the brand they last used. Past 30-day e-cigarette users who reported the brand names of e-cigarettes they usually/last used were included in this study, resulting in a sample size of N = 2569 (Figure S1).

### 2.2. Measures

#### 2.2.1. JUUL Use

Respondents who knew the brand of e-cigarettes they usually/last used were asked, “What brand of electronic nicotine product/electronic nicotine cartridge/e-liquid do/did you usually/last use?” Those who reported “JUUL” were coded as JUUL users, and those who reported other brands were coded as users of other brands.

#### 2.2.2. Past 30-Day E-Cigarette Consumption

E-cigarette consumption was measured as an estimation of the total puffs consumed in the past 30 days. Respondents were asked the average number of times they picked up e-cigarettes to take one or more puffs on the days they used and the number of puffs they took each time they picked up an e-cigarette to use. These two numbers were multiplied to obtain the number of puffs per day on the days e-cigarettes were used. To address outliers, daily puffs larger than 1000 were recoded to 1000 (i.e., the ceiling of daily puffs was set as 1000, which is the consumption of approximately 5 packs of 20-stick combustible cigarettes in puffs). Past 30-day e-cigarette consumption was then obtained as the multiplication of daily puffs and the number of days e-cigarettes were reported to have been used in the past 30 days.

#### 2.2.3. Past 30-Day E-Cigarette Use Frequency

E-cigarette use frequency was measured as the number of days respondents reported using e-cigarettes in the past 30 days at survey time. For better presentation and ease of interpretation of study results, past 30-day e-cigarette use frequency was categorized into six groups (1–2 days, 3–5 days, 6–9 days, 10–19 days, 20–29 days, and 30 days) for presenting in a figure and used as a continuous (1–30) covariate in the regression models.

#### 2.2.4. E-Cigarette Dependence Symptom and Other Covariates

This study used one measure to assess e-cigarette dependence, i.e., using e-cigarettes within 30 min after waking. Other covariates included in analyses were biological sex (male and female), race/ethnicity (non-Hispanic white, non-Hispanic Black, Hispanic, and other), parental education attainment for youth, and educational attainment for adults (less than high school, high school graduate, some college or associate degree, and bachelor’s degree or above), current cigarette smoking status (Yes/No), and current use of other tobacco products (Yes/No) based on findings from previous studies [38,39,40].

### 2.3. Analysis

Data management and analysis were conducted in October 2021 using Stata 16.1 (StataCorp, College Station, TX, USA). Wave 5 single-wave weights were applied to account for the complex sampling design and generate nationally representative estimates. The study sample was categorized into youth (12–17 years old, n = 390), young adults (18–24 years old, n = 1183), and adults (25 years old and above, n = 996) and analyzed separately. Descriptive statistics for the study sample were reported. Age compositions of JUUL use vs. use of other e-cigarette brands by prevalence and consumption amount (measured in puffs) were presented, along with age composition in the general population. Past 30-day e-cigarette use frequency of JUUL vs. other brands by age group was also reported. Multivariate logistic regressions were used to estimate the associations between JUUL use and related factors (e-cigarette use frequency and covariates listed above), and the association between e-cigarette dependence and JUUL use, controlling for e-cigarette use frequency and the covariates listed above, by age group.

## 3. Results

### 3.1. Study Sample

Among all respondents of the PATH study Wave 5 survey, 8.3% of youth, 26.2% of young adults, and 6.6% of adults were past 30-day e-cigarette users (results not shown in the table). Among past 30-day e-cigarette users, 37.7% of youth, 41.8% of young adults, and 39.2% of adults knew the brand name of e-cigarettes they usually/last used. Among those who knew the brand name, 65.2% of youth, 60.7% of young adults, and 25.6% of adults usually/last used JUUL (Figure S1).

### 3.2. Descriptive Statistics

Among youth participants in this study, 54.2% were male, 69.5% were non-Hispanic white, 40.1% had parents with a bachelor’s degree or above, 23.7% were current cigarette smokers, and 14.4% were current other tobacco users. Among young adult participants, 62.3% were male, 75.0% were non-Hispanic white, 10.7% had a bachelor’s degree or above, 29.8% were current cigarette smokers, and 25.3% were current other tobacco users. Among adult participants, 54.9% were male, 76.0% were non-Hispanic white, 20.7% had a bachelor’s degree or above, 52.8% were current cigarette smokers, and 22.6% were current other tobacco users (Table S1).

### 3.3. Use of JUUL vs. Other Brands by Prevalence and Consumption

**Figure 1:**
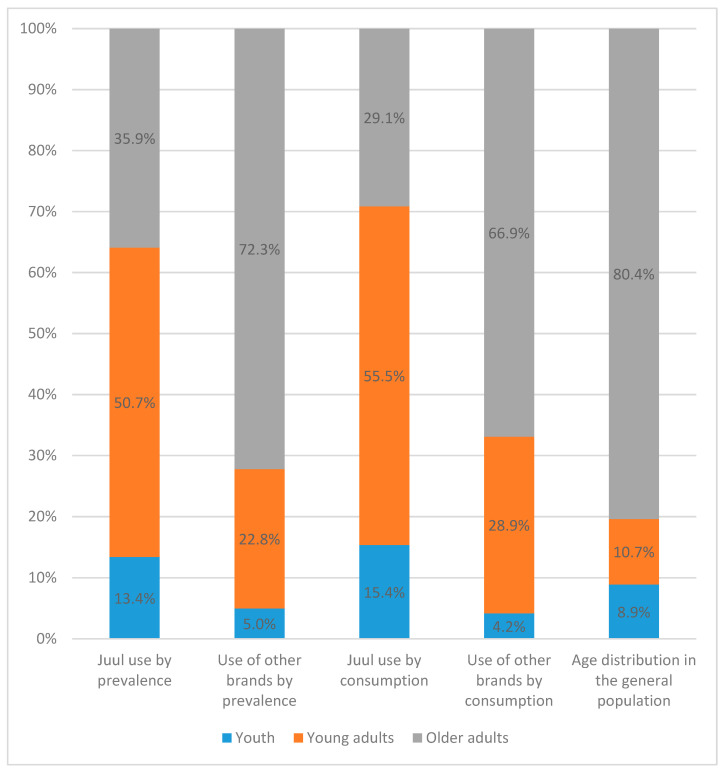
Distribution of JUUL use vs. use of other e-cigarette brands among U.S. youth, young adult, and adult e-cigarette users, compared with the proportion of population size.

The 2019 census data showed that among the U.S. population 12 years and older, about 9% were youth (age 12–17), 11% were young adults (age 18–24), and 80% were adults (25 and above) [41]. Among past 30-day e-cigarette users who reported JUUL as the brand of e-cigarettes they usually/last used, 13.4% were youth, 50.7% were young adults, and 35.9% were adults. Among users of other brands, 5.0% were youth, 22.8% were young adults, and 72.3% were adults. Of all past 30-day JUUL consumption measured in puffs, 15.4% was consumed by youth, 55.5% by young adults, and 29.1% by adults. By contrast, of consumption of other e-cigarette brands, 4.2%, 28.9%, and 66.9% were consumed by youth, young adults, and adults, respectively (Figure 1).

### 3.4. Use Frequency of JUUL vs. Other Brands by Age Group

**Figure 2:**
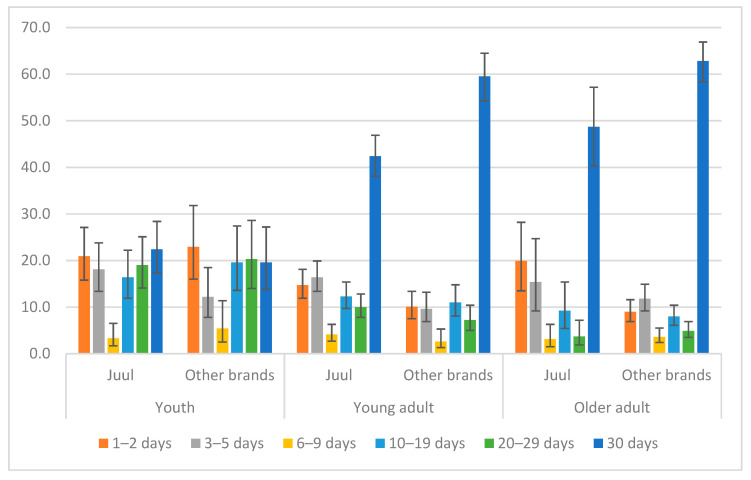
Percentages of JUUL users vs. users of other e-cigarette brands by use frequency and age group.

Past 30-day e-cigarette use frequency of JUUL vs. other brands by age group is presented in Figure 2. The distribution of use frequency among youth was similar between JUUL and other brands (JUUL vs. other brands: 20.9% vs. 22.9% for 1–2 days, 19.0% vs. 20.3% for 20–29 days, and 22.4% vs. 19.6% for daily use). Young adults and adults who used other e-cigarette brands were less likely to be rare users (1–2 days in the past 30 days) and more likely to be daily users than JUUL users. Among young adults, the use frequency of JUUL vs. other brands was 14.7% vs. 10.1% for 1–2 days, 16.5% vs. 9.6% for 3–5 days, and 42.4% vs. 59.5% for daily users. Among adults, the use frequency of JUUL vs. other brands was 19.9% vs. 9.0% for 1–2 days, 15.4% vs. 11.8% for 3–5 days, and 48.7% vs. 62.8% for daily users.

### 3.5. Factors Associated with JUUL Use by Age Group

Table 1 shows the estimated associations between JUUL use and e-cigarette use frequency, socio-demographic characteristics, and tobacco use status among past 30-day e-cigarette users. Among youth, current cigarette smokers were less likely to use JUUL (aOR = 0.55, 95% CI: 0.30–0.99). Young adults and adults who used e-cigarettes more frequently were less likely to use JUUL. Specifically, the estimated odds of JUUL use was 0.97 times for an additional one-day increase in past-30-day e-cigarette use among young adults (95% CI: 0.96–0.98) and adults (95% CI: 0.96–0.99). Additionally, young adults and adults with lower education levels were less likely to use JUUL than those with bachelor’s degrees or above. Compared with non-Hispanic whites, Hispanics were less likely to use JUUL among young adults, and non-Hispanic Blacks and non-Hispanic others were less likely to use JUUL among adults.

### 3.6. JUUL Use and E-Cigarette Dependence

Adjusted associations between e-cigarette dependence (measured as using e-cigarettes within 30 min after waking) and JUUL use are presented in Table 2. Youth JUUL users were more likely to use e-cigarettes within 30 min after waking (aOR = 2.30, 95% CI: 1.12–4.75), and adult JUUL users were less likely to use e-cigarettes within 30 min after waking (aOR = 0.57, 95% CI: 0.38–0.86), and this association was not significant among young adults. Past 30-day e-cigarette use frequency was significantly associated with using e-cigarettes within 30 min after waking, with an additional one-day increase in past 30-day use increased the odds about 1.1 times (aOR = 1.14, 95% CI: 1.10–1.18 for youth, aOR = 1.10, 95% CI: 1.08–1.13 for young adults, and aOR = 1.07, 95% CI: 1.05–1.09 for adults, respectively).

## 4. Discussion

This study used data from a nationally representative survey. It systematically examined the use patterns of JUUL vs. other e-cigarette brands among U.S. youth, young adult, and adult e-cigarette users. Findings from this study revealed that: (1) in 2019, JUUL was consumed disproportionately by youth and young adults, especially by young adults, in the U.S.; (2) JUUL use was popular among youth population subgroups regardless of socio-demographic factors, such as sex, race/ethnicity, and parental education level; (3) JUUL use was positively associated with e-cigarette dependence (i.e., use of e-cigarettes within 30 min of waking) among youth, and negatively associated with e-cigarette dependence among adults 25 years and older, compared with their counterparts who reported using other brands of e-cigarettes; (4) among youth e-cigarette users, those who did not smoke cigarettes were more likely to use JUUL e-cigarettes; and (5) adult e-cigarette users who reported using other brands used e-cigarettes more frequently than those who reported using JUUL.

This study is the first to estimate the age composition of JUUL use vs. the use of other e-cigarette brands. Consistent with previous studies [9,12,13], our results showed that JUUL was popular among youth and young adults, as 65.2% of youth and 60.7% of young adults who knew the brand names reported JUUL as the e-cigarettes they usually/last used in 2019. In addition, the share of JUUL consumed in the past 30 days, measured by the total number of puffs, was 15.4% by youth and 55.5% by young adults. In contrast, the share of other e-cigarettes consumed was 4.2% by youth and 28.9% by young adults. JUUL’s high popularity among youth and young adults, measured by consumption level and prevalence [3,6,42], calls for targeted efforts to communicate the health risks of nicotine addiction to youth and young adults. In addition, policies to strengthen the restrictions on underage access to e-cigarettes may be needed.

Our study also examined factors associated with JUUL use by age group among current e-cigarette users. Key findings include differences across certain racial/ethnic groups and education levels among young adult and adult e-cigarette users. In addition, we found that JUUL use was not associated with socio-demographic factors among youth e-cigarette users, indicating that JUUL was universally popular among youth population subgroups characterized by socio-demographic factors compared with other e-cigarette brands. Given the popularity of JUUL e-cigarettes among youth across all youth subgroups [3,6,42], this finding is not completely unexpected. The differences characterized by socioeconomic status (SES) observed among young adult and adult e-cigarette users may be partially explained by the fact that individuals with higher SES may be more likely to choose higher price tobacco products with better aesthetic design and features [43]. For youth e-cigarette users, their brand choice may be more affected by product marketing [44,45,46], particularly online and social media marketing [47,48].

Additionally, this study estimated the association between JUUL use and e-cigarette dependence, measured as the use of e-cigarettes within 30 min of waking. Specifically, we found that youth JUUL users were more likely to use e-cigarettes within 30 min of waking than their counterparts who used other e-cigarette brands. By contrast, adult JUUL users were less likely to use e-cigarettes within 30 min of waking. Previous studies found that JUUL use was associated with greater odds of nicotine dependence among U.S. adolescents [35,49]. Evidence documents that JUUL delivers nicotine substantially faster than most other e-cigarette brands [22], which may lead to greater symptoms of nicotine dependence. In previous studies, nicotine dependence was measured as the craving to use any tobacco product and/or use a tobacco product within 30 min of waking [35,49]. Our study extended the analysis to adults and specifically measured participants’ dependence on e-cigarettes. Given the elevated odds of e-cigarette dependence among youth JUUL users compared with other e-cigarette brands, tobacco and nicotine cessation interventions for youth may need to consider incorporating specific efforts targeted at JUUL users.

Furthermore, this study revealed that youth e-cigarette users who were not currently smoking cigarettes were more likely to use JUUL e-cigarettes than other e-cigarette brands compared with those who were current cigarette smokers. However, among young adult and adult e-cigarette users, cigarette smoking was not associated with either increased or decreased odds of using JUUL e-cigarettes vs. other brands of e-cigarettes. These findings seem to be inconsistent with JUUL’s claim that their product is an “alternative to smoking” to help “transition the world’s billion adult smokers away from combustible cigarettes” [50]. However, because this is not a longitudinal study, it is unclear whether JUUL served as an off-ramp for smoking or an initial nicotine source for youth users.

Our study also found that the use frequency—measured as the number of days using e-cigarettes in the past 30 days—of JUUL and other e-cigarette brands were different across age groups, a finding not reported in previous studies. Both bivariate and adjusted associations showed that JUUL use was associated with less frequent e-cigarette use among young adult and adult users. This indicates that although JUUL was one of the most used e-cigarette brands among adults in the US, they were used less frequently than other brands of e-cigarettes. This may have implications on the role of JUUL e-cigarettes in smoking cessation, given the findings from the previous studies, which have shown that frequent e-cigarette use among smokers was associated with a higher likelihood of smoking cessation [39,51,52].

Findings from this study could provide important evidence to help inform current regulatory decisions related to JUUL. FDA’s regulatory decisions have indicated a concern that JUUL is marketing to teenagers and contributing to youth vaping [33]. In this study, we found that JUUL was consumed disproportionately by youth and young adults. Pressed by federal and state investigations, JUUL suspended most of its social media marketing activities and withdrew flavored JUUL pods (except for menthol) in 2018/2019. However, our results showed that more than 70% of JUUL e-cigarettes were still consumed by youth and young adults in 2019.

This study has limitations. First, if participants reported JUUL (or other brands) to be the e-cigarettes they usually/last used, then we assumed they used this brand of e-cigarettes exclusively in the past 30 days. In addition, a large proportion of past 30-day e-cigarette users (about 60%) in the PATH study did not know the brand names of e-cigarettes they frequently/last used. Therefore, the estimated consumption puffs and use frequency for JUUL and other e-cigarette brands may be different when those who did not report their usual/last used e-cigarette brands were considered. Furthermore, this study used self-reported data, which may be subject to recall bias and social desirability bias. Additionally, the PATH Wave 5 data were collected from December 2018 to November 2019, which may not accurately reflect the current consumption proportion of JUUL vs. other brands of e-cigarettes. It is also worth noting that the data was collected before the outbreak of COVID-19, which may have a substantial impact on the e-cigarette marketplace and use behaviors [53,54,55]. Furthermore, this study only used one indicator to measure e-cigarette dependence, which may be insufficient. However, there are still no established standards for assessing e-cigarette dependence [56]. Future studies could use more advanced modeling methodologies to measure e-cigarette dependence from multiple dimensions. Finally, our study relied on the data from the PATH survey. Previous studies have shown that the estimated prevalence of e-cigarette use was lower among youth and higher among young adults in the PATH survey compared with the estimates from other nationally representative surveys [9,57,58]. As such, the consumption level in our study may be under-estimated among youth but over-estimated among young adults.

## 5. Conclusions

This study found that compared with other e-cigarette brands, JUUL was consumed disproportionately by youth and young adults, especially by young adults, in the U.S. Adult e-cigarette users who reported using JUUL used e-cigarettes less frequently than those who reported using other brands of e-cigarettes. Youth e-cigarette users who did not smoke cigarettes were more likely to use JUUL than those who currently smoked cigarettes. Additionally, while the adult (25+) JUUL users were less likely to use e-cigarettes within 30 min after waking, youth JUUL users appeared to be more likely to use e-cigarettes within 30 min after waking. More efforts are needed to prevent e-cigarette initiation and nicotine addiction among U.S. youth and young adults.

## Figures and Tables

**Table 1:** Associations between JUUL use and e-cigarette use frequency, socio-demographic characteristics, and other tobacco use among U.S. youth, young adult, and adult e-cigarette users.

Individual Characteristics	Youth		Young Adults		Adults	
	OR	95% CI	OR	95% CI	OR	95% CI
E-cigarette use frequency	1.01	0.99–1.03	0.97	0.96–0.98	0.97	0.96–0.99
Sex						
Male	0.63	0.39–1.03	0.99	0.74–1.32	1.39	0.93–2.07
Female	Ref		Ref		Ref	
Race/ethnicity						
Non-Hispanic White	Ref		Ref		Ref	
Non-Hispanic Black	0.55	0.15–2.03	1.29	0.71–2.33	0.38	0.16–0.91
Hispanic	0.67	0.37–1.23	0.64	0.42–0.98	0.76	0.40–1.44
Non-Hispanic other	1.18	0.54–2.57	0.77	0.44–1.33	0.31	0.13–0.75
Education/Parental education						
Less than high school	0.58	0.26–1.26	0.29	0.15–0.57	0.28	0.15–0.55
High school graduate	0.49	0.22–1.09	0.34	0.18–0.64	0.42	0.23–0.78
Some college or associate degree	0.63	0.35–1.12	0.54	0.29–0.99	0.48	0.30–0.74
Bachelor’s degree or above	Ref		Ref		Ref	
Cigarette smoking						
Yes	0.55	0.30–0.99	0.85	0.62–1.16	1.16	0.77–1.74
No	Ref		Ref		Ref	
Other tobacco use						
Yes	0.54	0.26–1.11	0.45	0.32–0.63	0.75	0.45–1.24
No	Ref		Ref		Ref	

**Table 2:** Association between e-cigarette dependency (measured as using e-cigarette within 30 min after waking) and JUUL use among U.S. youth, young adults, and adults.

Individual Characteristics	Youth		Young Adults		Adults	
	OR	95% CI	OR	95% CI	OR	95% CI
JUUL user						
Yes	2.30	1.12–4.75	1.17	0.84–1.64	0.57	0.38–0.86
No	Ref		Ref		Ref	
E-cigarette use frequency	1.14	1.10–1.18	1.10	1.08–1.13	1.07	1.05–1.09
Sex						
Male	1.18	0.63–2.23	1.29	0.91–1.84	0.72	0.51–1.01
Female	Ref		Ref		Ref	
Race/ethnicity						
Non-Hispanic White	Ref		Ref		Ref	
Non-Hispanic Black	1.18	0.28–5.07	0.47	0.21–1.06	0.85	0.45–1.63
Hispanic	1.09	0.47–2.54	0.77	0.47–1.27	0.48	0.25–0.93
Non-Hispanic other	0.99	0.28–3.50	0.67	0.37–1.23	0.48	0.24–0.97
Education/Parental education						
Less than high school	0.96	0.27–3.43	1.25	0.60–2.59	1.65	0.95–2.87
High school graduate	1.69	0.72–3.96	1.30	0.68–2.48	1.17	0.70–1.98
Some college or associate degree	0.80	0.37–1.76	0.97	0.51–1.85	1.21	0.77–1.90
Bachelor’s degree or above	Ref		Ref		Ref	
Cigarette smoking						
Yes	2.02	0.97–4.18	1.12	0.76–1.65	0.70	0.49–1.01
No	Ref		Ref		Ref	
Other tobacco use						
Yes	1.90	0.61–5.91	1.56	1.07–2.28	1.23	0.80–1.89
No	Ref		Ref		Ref	

## Data Availability

Publicly available datasets were analyzed in this study. This data can be found here: [https://www.icpsr.umich.edu/web/NAHDAP/studies/36231 (accessed on 16 May 2022)].

## Supplementary Materials

The following supporting information can be downloaded at: https://www.mdpi.com/article/10.3390/ijerph191710837/s1, Figure S1: Proportion of respondents who knew the e-cigarette brand names they frequently/last used among U.S. youth, young adult, and adult past 30-day e-cigarette users in 2019 (PATH Wave 5); Table S1: Descriptive statistics of past 30-day e-cigarette users who know the brand names they usually/last used among U.S. youth, young adults, and adults.
