# Supplementary material for: Consumption of JUUL vs. Other E-Cigarette Brands among U.S. E-Cigarette Users: Evidence from Wave 5 of the PATH Study
Source: Int J Environ Res Public Health. 2022 Aug 31;19(17):10837. doi: 10.3390/ijerph191710837 (PMC9518567; doi:10.3390/ijerph191710837)
Supplement: Supplementary file 1 [file ijerph-19-10837-s001.zip › ijerph-1867708-supplementary.pdf]

Figure S1. Proportion of respondents who knew the e-cigarette brand names they frequently/last used among U.S. youth, young adult, and adult past 30-day e-cigarette users in 2019 (PATH Wave 5).

1a. Youth (Unweighted frequency: 390 youth reported the brand names of e-cigarette they usually/last used, 243 reported JUUL.)

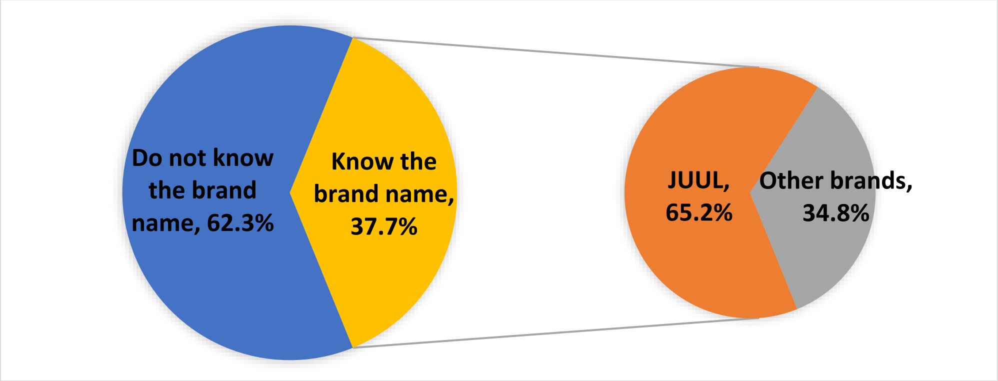

1b. Young adults (Unweighted frequency: 1,183 young adults reported the brand names of e-cigarette they usually/last used, 699 reported JUUL)

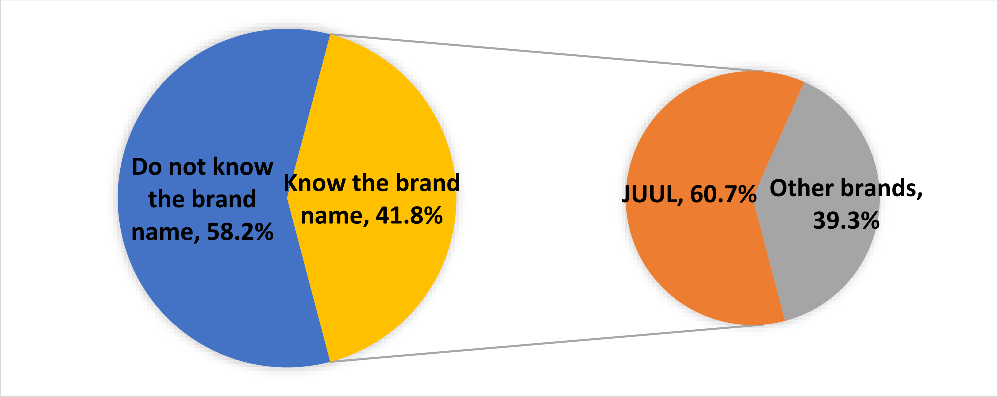

1c. Adults (Unweighted frequency: 996 adults reported the brand names of e-cigarette they usually/last used, 238 reported JUUL)

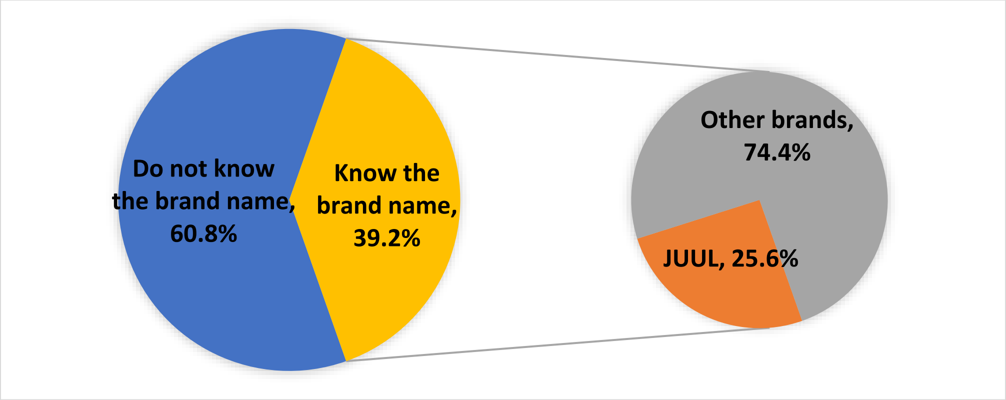

Table S1. Descriptive statistics of past 30-day e-cigarette users who know the brand names they usually/last used among U.S. youth, young adults, and adults.

	Youth		Young adults		Adults	
Individual characteristics	**%**	95% CI	**%**	95% CI	**%**	95% CI
Sex						
Male	54.2	48.9 - 59.5	62.3	59.1 - 65.4	54.9	51.0 - 58.7
Female	45.8	40.5 - 51.1	37.7	34.6 - 40.9	45.1	41.3 - 49.0
Race/ethnicity						
Non-Hispanic White	69.5	64.4 - 74.1	75.0	72.2 - 77.7	76.0	72.8 - 79.0
Non-Hispanic Black	3.0	1.7 - 5.2	5.1	3.9 - 6.6	7.9	6.1 - 10.1
Hispanic	17.7	14.1 - 22.0	11.7	10.0 - 13.7	9.4	7.6 - 11.6
Non-Hispanic other	9.8	7.0 - 13.5	8.2	6.5 - 10.3	6.7	5.1 - 8.6
Education/Parental education						
Less than high school	11.3	8.4 - 15.0	13.7	11.7 - 15.9	14.4	12.2 - 17.0
High school graduate	15.9	12.3 - 20.2	28.3	25.6 - 31.1	26.9	23.2 - 30.9
Some college or associate degree	32.8	28.0 - 37.9	47.4	44.2 - 50.7	37.9	34.5 - 41.6
Bachelor's degree or above	40.1	34.9 - 45.5	10.7	8.6 - 13.1	20.7	17.9 - 23.9
Cigarette smoking						
Yes	23.7	19.5 - 28.4	29.8	26.9 - 32.9	52.8	48.9 - 56.7
No	76.3	71.6 - 80.5	70.2	67.1 - 73.1	47.2	43.3 - 51.1
Other tobacco use						
Yes	14.4	11.0 - 18.8	25.3	22.6 - 28.3	22.6	19.5 - 26.0
No	85.6	81.2 – 89.0	74.7	71.7 - 77.4	77.4	74.0 - 80.5
